# Lactitol Supplementation Modulates Intestinal Microbiome in Liver Cirrhotic Patients

**DOI:** 10.3389/fmed.2021.762930

**Published:** 2021-10-14

**Authors:** Haifeng Lu, Liang Chen, Xiaxia Pan, Yujun Yao, Hua Zhang, Xiaofei Zhu, Xiaobin Lou, Chunxia Zhu, Jun Wang, Lanjuan Li, Zhongwen Wu

**Affiliations:** ^1^State Key Laboratory for Diagnosis and Treatment of Infectious Diseases, First Affiliated Hospital, School of Medicine, Zhejiang University, Hangzhou, China; ^2^National Clinical Research Center for Infectious Diseases, First Affiliated Hospital, School of Medicine, Zhejiang University, Hangzhou, China; ^3^CAS Key Laboratory of Pathogenic Microbiology and Immunology, Institute of Microbiology, Chinese Academy of Science, Beijing, China; ^4^University of Chinese Academy of Sciences, Beijing, China

**Keywords:** cirrhosis, lactitol, microbiome, short-chain fatty acids, bile acids, shotgun metagenomics, targeted metabolomics

## Abstract

**Background:** Cirrhosis is a common chronic liver disease characterized by irreversible diffuse liver damage. Intestinal microbiome dysbiosis and metabolite dysfunction contribute to the development of cirrhosis. Lactitol (4-β-D-galactopyranosyl-D-glucitol) was previously reported to promote the growth of intestinal *Bifidobacteria*. However, the effect of lactitol on the intestinal microbiome and fecal short-chain fatty acids (SCFAs) and bile acids (BAs) and the interactions among these factors in cirrhotic patients pre- and post-lactitol treatment remain poorly understood.

**Methods:** Here, using shotgun metagenomics and targeted metabolomics methods.

**Results:** we found that health-promoting lactic acid bacteria, including *Bifidobacterium longum, B.pseudocatenulatum*, and *Lactobacillus salivarius*, were increased after lactitol intervention, and significant decrease of pathogen *Klebsiella pneumonia* and associated antibiotic resistant genes /virulence factors. Functionally, pathways including Pseudomonas aeruginosa biofilm formation, endotoxin biosynthesis, and horizontal transfer of pathogenic genes were decreased in cirrhotic patients after 4-week lactitol intervention compared with before treatment.

**Conclusion:** We identified lactitol-associated metagenomic changes, and provide insight into the understanding of the roles of lactitol in modulating gut microbiome in cirrhotic patients.

## Introduction

Cirrhosis is a common chronic liver disease characterized by irreversible diffuse liver damage. It is an important risk factor for hepatocellular carcinoma and hepatic decompensation development. The early diagnosis and treatment of cirrhotic patients are essential to achieve the best outcomes. Antiviral drug treatments fail to effectively prevent the progression to liver cirrhosis or its associated complications, which suggests that hepatitis virus replication is not the only driving force of cirrhosis development in China (1, 2). The intestinal microbiota was demonstrated to be associated with the pathogenesis and progression of liver diseases, including alcoholic liver disease (3), nonalcoholic fatty liver disease, non-alcoholic steatohepatitis (4), total parenteral nutrition/intestinal failure-related liver disease (5), and primary sclerosing cholangitis (PSC) (6). Moreover, there are direct interactions between metabolites produced by the gut microbiota and cirrhosis (7). In particular, the intestinal microbiota is a topic of high interest because an increasing number of researchers have focused their efforts on the potential roles of intestinal microbiota-targeted therapies in ameliorating the progression of liver disease (8, 9). Furthermore, it has been suggested that therapies targeting the intestinal microbiome (10) are pivotal for the treatment of liver fibrosis and cirrhosis.

Lactitol (4-beta-D-galactopyranosyl-D-glucitol), as a prebiotic, can be metabolized by intestinal bacteria to lactic acid and other natural acids. The efficacy of lactitol in the treatment of constipation and hepatic encephalopathy has been evaluated in various clinical trials (11–14). Lactitol supplementation can effectively increase the abundance of beneficial intestinal bacterial communities, such as *Bifidobacterium* and *Lactobacillus*. Moreover, lactitol decreased the levels of plasma endotoxin compared with other standard medical treatments, such as antibiotics (15). Although other alterations in the interactions between the microbiota and metabolites after the oral administration of lactitol have not been clearly and comprehensively elucidated, lactitol is widely used in the clinic treatment of portal-systemic encephalopathy to reduce liver injury through regulating the intestinal microbial community and decreasing gut-derived endotoxemia (12, 13). Therefore, it is important to understand the physiological mechanisms of the intestinal microbiome and metabolites associated with lactitol supplementation, and identify microbiome involved in benefiting patients and clinical outcomes.

In this study, we provided insight into benefits of lactitol to cirrhotic disease by profiling of intestinal microbiome and metabolites of cirrhotic patients before and after 4 weeks lactitol treatment. Compared with healthy individuals, we examined the correlation between the microbiome and metabolites and found shifts of the abundance of microbial antibiotic-resistant genes (ARGs), virulence factor genes (VFGs) in microbiome, and metabolites including short-chain fatty acids (SCFAs) and bile acids (BAs).

## Patients and Methods

### Subjects and Study Design

Subjects with histologically confirmed liver cirrhosis and a body mass index (BMI) <25.78 kg/m2 were classified as the lactitol group (LC). Clinical, metabolic biochemical, and epidemiological characteristics are described in Supplementary Table 1. This study was performed in accordance with the ethical guidelines of the 1975 Declaration of Helsinki for the participation of human subjects and approved by the Ethics Committee of the First Affiliated Hospital, School of Medicine, Zhejiang University (reference number: 2017HL668-1). Written informed consent and questionnaires were obtained from all study subjects at the time of enrolment. The inclusion and exclusion criteria were presented in Supplementary File 1. Finally, the study included 24 patients and 29 healthy controls (HCs). The details of these subjects are presented in Table 1. In the patient group, lactitol was administered orally at a dose of 5 g three times a day, and samples were collected after 4 weeks of treatment and subjected to intestinal microbiome metagenomics and metabolomics analyses.

**Table 1:** Subject characteristics.

Characteristics		**Group HC**	**Group LC_pre**	**Group LC_post**	P-value		
		**N=29 (%)**	N=24 (%)		**LC_pre vs HC**	**LC_post vs HC**	**LC_ post vs LC_ pre**
Gender	Female	8 (27.58)	7 (29.17)		0.840	0.840	–
	Male	21 (72.42)	17 (70.83)				
Age		50.77 ± 6.76	51.58 ± 10.95		0.751	0.751	–
BMI (kg/m2)		21.58 ± 3.05	22.30 ± 2.74		0.370	0.370	–
ALT (5-40U/L)		25.23 ± 10.46	26.83 ± 12.23	31.08 ± 18.11	0.678	0.132	0.297
AST (8-40U/L)		24.97 ± 6.52	34.54 ± 14.55	34.83 ± 14.98	0.007	0.005	0.936
Albumin (35-55g/L)		45.34 ± 2.71	41.96 ± 4.68	43.24 ± 4.85	0.012	0.196	0.744
Globulin (20-35g/L)		26.27 ± 2.98	30.31 ± 5.43	29.98 ± 5.15	0.009	0.012	0.995
TB (0-21mg/dL)		11.37 ± 5.29	25.08 ± 14.70	24.87 ± 11.82	<0.001	<0.001	1.000
PLT(83-303*109/L)		233.83 ± 49.99	84.71 ± 56.69	85.08 ± 57.44	<0.001	<0.001	1.000
Crea(59-104mg/dL)		73.50 ± 11.96	69.54 ± 11.97	66.42 ± 11.31	0.232	0.034	0.370
INR		–	1.18 ± 0.14	1.17 ± 0.11	–	–	1.000
MELD		–	5.37 ± 0.34	5.34 ± 0.30	–	–	0.984

*The data were depicted as Mean ± SD. Continuous variables with a normal distribution were assessed using two-tailed independent sample t tests, whereas the data that did not fit a normal distribution were assessed using a non-parametric Mann-Whitney test*.

### Biological Specimen Collection and DNA Extraction

Well-formed stools were collected in sterile bags, refrigerated, and then taken directly to the lab. The samples were then divided into 200-mg aliquots, frozen rapidly in liquid nitrogen and stored at −80° until DNA extraction. Three blood samples were collected in tubes (two tubes without additives and one EDTA-containing tube) labeled with the subject's study number and delivered immediately to our clinical diagnostics laboratory for routine blood tests biochemical parameter measurements, and serum collection. Serum samples were divided into 100-μL aliquots and stored at −80°C until BA quantification. Samples (including stools and peripheral blood samples) were collected from cirrhotic patients at two time points: enrolment and 4 weeks after lactitol treatment (5 g, three times a day). HCs provided their samples only once at the time of enrollment.

Fecal DNA was extracted from the fecal pellets with a QIAGEN DNeasy PowerSoil Kit (ref. no.12888, Germany) in accordance with the manufacturer's instructions. The DNA samples were quantified with a Qubit dsDNA HS Assay Kit (Invitrogen, Q32854), normalized to 20 ng/L, and stored at −80°C until sequencing library preparation.

### Metagenomic Sequencing and Analysis

For this assay, 10 L of DNA were fragmented by sonication to a size of 350 bp using an Ultrasonic 5-cell crusher (Covaris LE220R-plus, USA). An NEB Next^Ⓡ^ UltraTM DNA library Prep Kit for Illumina (NEB, USA) was used to generate the DNA sequencing library, and an attribute sequence index code was added to each fecal DNA sample. The library quality was assessed using the Qubit^Ⓡ^ 2.0 Fluorometer (Life Technologies, CA, USA) and Agilent Bioanalyzer 2100 system. Sequencing was performed on an Illumina Novaseq 6000 platform (Illumina) using NovaSeq 6000 Reagent Kits v1.5 (Illumina, USA) in accordance with the manufacturer's instructions. Finally, 150-bp paired-end reads were generated, and the raw sequencing data were submitted to the National Microbiology Data Center. The accession number is NMDC10017859.

Raw sequencing reads were quality-controlled with KneadData (version 0.6.1, https://bitbucket.org/biobakery/kneaddata). Briefly, low-quality bases were trimmed from the 3′ end of reads with Trimmomatic (version 0.36) (16), and then trimmed reads <50 nucleotides in length were discarded. Host (human) contaminant reads were identified and removed by mapping against the human genome (hg19) with Bowtie 2 (17). Quality-filtered reads were taxonomically profiled using MetaPhlAn2 (version 2.2.0) (18) against the ChocoPhlAn database, and community-level functional metabolic pathways were identified using HUMAnN2 (19) against the UniRef90 protein reference database with default settings. The resulting taxonomic and pathway abundance files from all samples were joined and normalized to the relative abundance. Merged data were unstratified, and LEfSe (20) was used to identify differentially abundant features, as described above.

To explore the metagenomic information of the intestinal microbiota, all quality-filtered reads from samples were co-assembled using MEGAHIT (v.1.0.3) (21), and potential genes were predicted over the assembled contigs by Prokka (22). Genes with a length greater than or equal to 100 bp were retained. All predicted genes were combined and clustered using CD HIT (23) into a non-redundant gene set by setting the similarity threshold to 95%, and the relative abundance of unique genes was calculated using SALMON (24). Then, the predicted genes were annotated using eggNOG 4.5 (25), and Clusters of Orthologous Group (COG) (26) profiles were generated. Furthermore, all protein predictions were searched against the CARD database using resistance gene identifier software (27) and the VFDB database (28) using DIAMOND (29). Additional details are presented in Supplementary File 1.

### BA Quantification

Samples (20 mg for each fecal sample and 50 μL for each blood sample) were extracted with 200 μL methanol after being ground with a ball mill, and the proteins were precipitated at −20°. The extracts were evaporated to dryness and reconstituted in 100 μL 50% methanol (V/V) for further LC-MS analysis using an LC-ESI-MS/MS system (UHPLC, ExionLC^TM^ AD; MS, Applied Biosystems 6500 Triple Quadrupole). Details were presented in Supplementary File 1. The standards, including cholic acid-d4, lithocholic acid-d4, glycolithocholic acid-d4, glycochenodeoxycholic acid-d4, taurocholic acid-d4, tauroursodeoxycholic acid-d5, glycodeoxycholic acid-d4, chenodeoxycholic acid-d4, deoxycholic acid-d4, and 2-chloro-L-phenylalanine, were purchased from Olchemim Ltd. (Olomouc, Czech Republic) and Sigma (St. Louis, MO, USA). A set of diluted standards (1000 μg/mL, 100 μg/mL, 10 μg/mL, 1 μg/mL, 0.1 ^μ^g/mL, and 0.01 ^μ^g/mL) was prepared with MeOH before analysis. BA concentrations were depicted as nanograms per gram (ng/g) of feces in fecal samples and nanograms per milliliter (ng/mL) of blood in blood samples. Data analysis was performed with MetWare (http://www.metware.cn/) based on the AB SciexQTRAP 6500 LC-MS/MS platform.

### SCFA Quantification

Stool SCFAs were measured using a gas chromatography-mass spectrometry (GC-MS; Gas chromatograph (type 7890A, Agilent, USA) equipped with a mass selective detector (type 5975C, Agilent, USA). The measurement details were described in our previous study (30) and Supplementary File 1. The standards, including acetate-d3, propionate-d5, butyrate-d7, valerate-d9, isobutyric acid, 2-methylbutyric acid, and 3-methylbutyric acid (isovaleric acid), were purchased from Sigma (St. Louis, MO, USA). A set of diluted standards was prepared (1,000 μg/mL, 100 μg/mL, 10 μg/mL, 1 μg/mL, 0.1 μg/mL, and 0.01 μg/mL) with ethyl acetate before analysis. Data analysis was performed with ChemStation (Agilent, CA, USA, v E.02.02.1432) and Chroma TOF software (LECO, St. Joseph, MI, USA, v 4.34). SCFA concentrations in fecal samples were reported in milligrams per gram (mg/g) of feces.

### Statistical Analysis

All statistical analyses were performed using R software. Data are presented as the mean ± standard error of the mean (SEM). The Kruskal-Wallis sum test was used for comparisons between groups when appropriate. Bray-Curtis distance was calculated as the beta diversity measurement using the vegan package. The PERMANOVA test with the adonis function was used to calculate the community structure differences. Principal coordinate analysis was carried out to visualize the Bray-Curtis dissimilarity among samples for microbial species (Hellinger transformed). The different treatments were fitted as centroids onto the ordination plots (31). Different genes were identified using the DESeq2 package with the cutoff threshold of adjusted P < 0.05 and the absolute value of log2 fold change (log2 FC) > 1. KEGG enrichment analyses (http://www.genome.jp/kegg) were performed using different genes as the foreground genes and all genes as the background. Discriminatory metabolites between groups were determined by VIP ≥ 1 and fold change ≥ 2 or ≤ 0.5. VIP values were extracted from OPLS-DA results, which included score plots and permutation plots generated using R package MetaboAnalystR. The data were log-transformed (log2) and mean-centered before OPLS-DA. To avoid overfitting, a permutation test (200 permutations) was performed. Significantly enriched KEGG pathways were identified for the differentially expressed genes identified above with the FDR multiple testing-corrected P < 0.05. All figures were created using the ggplot2 and DEseq2 packages. P < 0.05 or BH-adjusted P < 0.05 was considered statistically significant.

## Results

### Baseline Characteristics of Subjects

Table 1 presents the detailed characteristics of each group, including clinical, metabolic, biochemical, and histological profiles. The levels of albumin, globulin and platelet in subjects with liver cirrhosis were lower than in the HC group. All clinical parameters including MELD showed no difference between pre- and post-lactitol treatment groups. During lactitol therapy, none of the patients experienced abdominal pain, bloating, diarrhea and other uncomfortable clinical manifestations.

### Compositional Alterations in the Gut Microbiome

Each sample was sequenced at an average 12G depth of data and contained 3,199,274 non-redundant ORFs. A total of 387 bacterial species were identified across all subjects and classified into 122 genera (>99% in each sample; Supplementary Materials 1A,B) for subsequent analysis.

**Figure 1:**
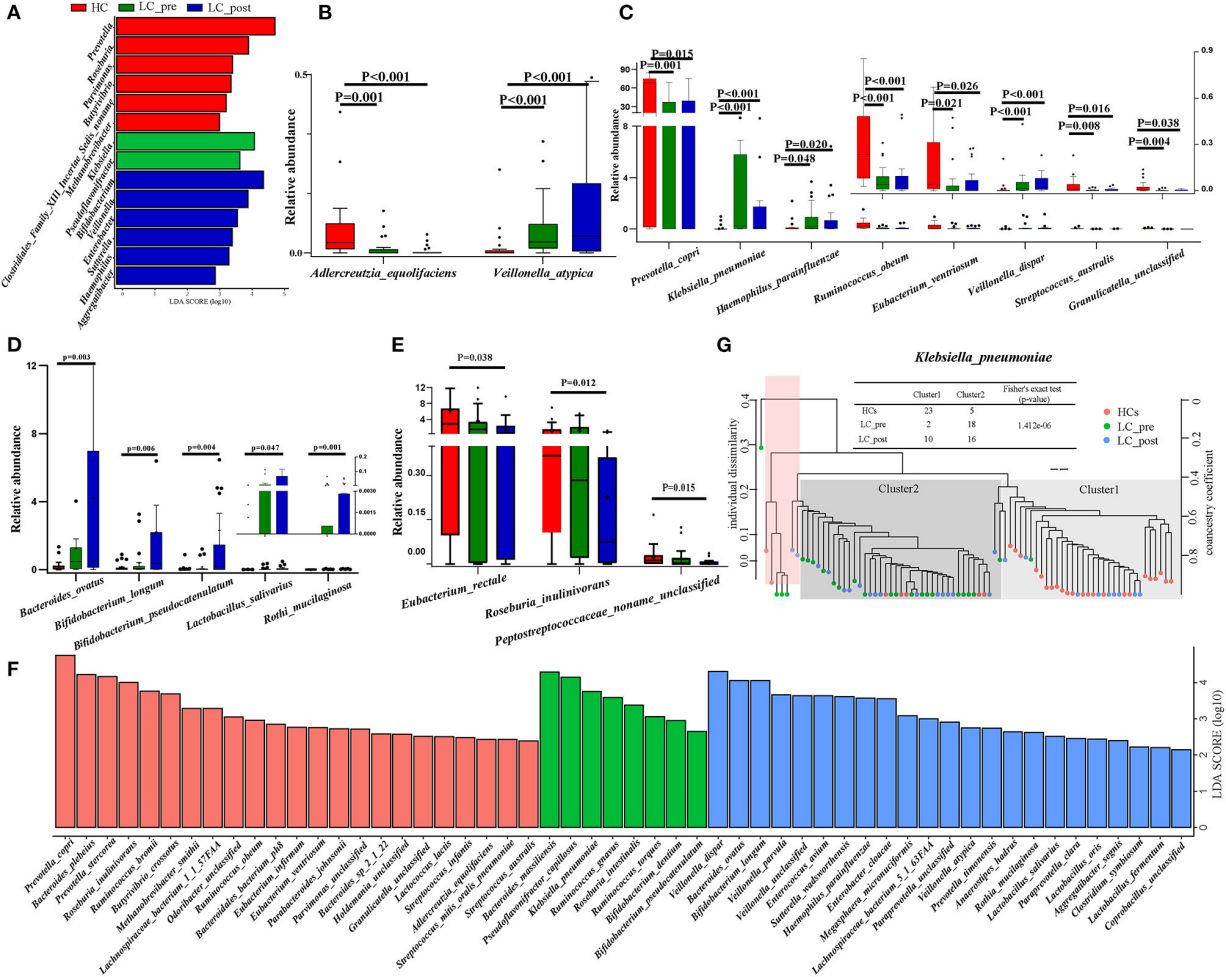
Comparison of intestinal microbial communities in cirrhotic patients before (LC-pre, green) and after (LC-post, blue) lactitol intervention and healthy controls (HCs, red). **(A)** LEfSe and LDA based on divergent genera between the three groups, which identified the most differentially abundant genera between the three groups. LDA scores of red bars represented genera enriched in HCs, the green represented the LC-pre group, and the blue represented the LC-post group. **(B–E)** Statistical analysis of differentially abundant species by a non-parametric Kruskal–Wallis test with the Bonferroni correction between the three groups. The box plots indicated the median and 25th to 75th percentiles. **(F)** LEfSe and LDA based on divergent species between the three groups. **(G)** Strain-level analysis of *K. pneumoniae* based on SNPs using the fecal metagenome of subjects in the three groups. LEfSe, Linear discriminant analysis Effect Size; LDA, linear discriminant analysis.

Although no significant differences were observed in α and β bacterial community diversities between patients and HCs or pre-/post-lactitol treatment groups (Supplementary Figures 1A–F; all *P* > 0.05), significant differences in abundances of bacterial taxa were found in the intestinal microbiota of the three groups. Detailed overviews of the dominant bacterial profiles in each group were illustrated at the genus and species level (Supplementary Figures 2A,B). Among these 20 dominant genera, *Bifidobacterium, Veillonella, Enterobacter, Sutterella, Haemophilus*, and *Aggregatibacter* were found to be enriched in LC-post group, whereas *Klebsiella* and *Pseudoflavonifractor* were enriched in the LC-pre group, and *Prevotella, Roseburia, Parvimonas, Butyrivibrio, Methanobrevibacter*, and Clostridiales XIII_incertae_sedis_noname were enriched in HCs (all LDA scores (log10) > 2 and *P* < 0.05, using the LefSe approach; Figure 1A). At the species level, *Adlercreutzia_equolifaciens* (lactitol-decreased) and *Veillonella_atypica* (lactitol-increased) were found to be further deepened the discrepancy in their abundance after 4 weeks of lactitol supplementation (Figure 1B, by Kruskal–Wallis test along with the Bonferroni correction); Furthermore, the effects of lactitol treatment on *Haemophilus_parainfluenzae, Ruminococcus_obeum*, and *Eubacterium_ventriosum* were minimal, and the shifts in the other species that were significantly discriminatory between the LC-pre vs. HC groups tended to be similar to the level of those in HCs, including three increased species (*Prevotella_copri, Streptococcus_australis*, and *Granulicatella*_unclassified) and two decreased species (*Klebsiella_pneumoniae* and *V. dispar*) (Figure 1C). Species that showed no difference in abundance between LC-pre and HC groups exhibited a significant difference in abundance between LC-post and HC groups, including five increased species (*Bacteroides_ovatus, Bifidobacterium_pseudocatenulatum, B. longum, Lactobacillus_salivarius*, and *Rothi_mucilaginosa* (Figure 1D) and three decreased species (*Peptostreptococcaceae*_noname_unclassified, *Roseburia_inulinivorans* and *Eubacterium_rectale* (Figure 1E). Additionally, we found some health-beneficial species enriched in the LC-post group (including *B. ovatus, B. pseudocatenulatum, B. longum, Lactobacillus_salivarius, L. fermentium*, and *L. oris*), while some opportunistic pathogen enriched in the LC-pre group (including *B. massiliensis, K. pneumoniae, R. onavus, R. torques* and *V. dispar*) (all LDA scores (log10) > 2 and *P* < 0.05; Figure 1F). Phylogenic trees based on single-nucleotide polymorphisms were constructed for these discriminatory species, and two distinct clusters were formed for most cirrhotic patients and HCs based on the genome of *K. pneumoniae* (*P* < 0.001, Fisher's exact test; Figure 1G). Interestingly, there was a significant shift in the genotypes of *K. pneumoniae* strains as 10 LC-post patient strains were clustered together with HC strains, suggesting that liver cirrhotic patients are colonized by different *K. pneumonia* strains compared with HCs, which were partially changed by lactitol treatment.

### Lactitol Treatment Modulated Microbial Functional Genes

**Figure 2:**
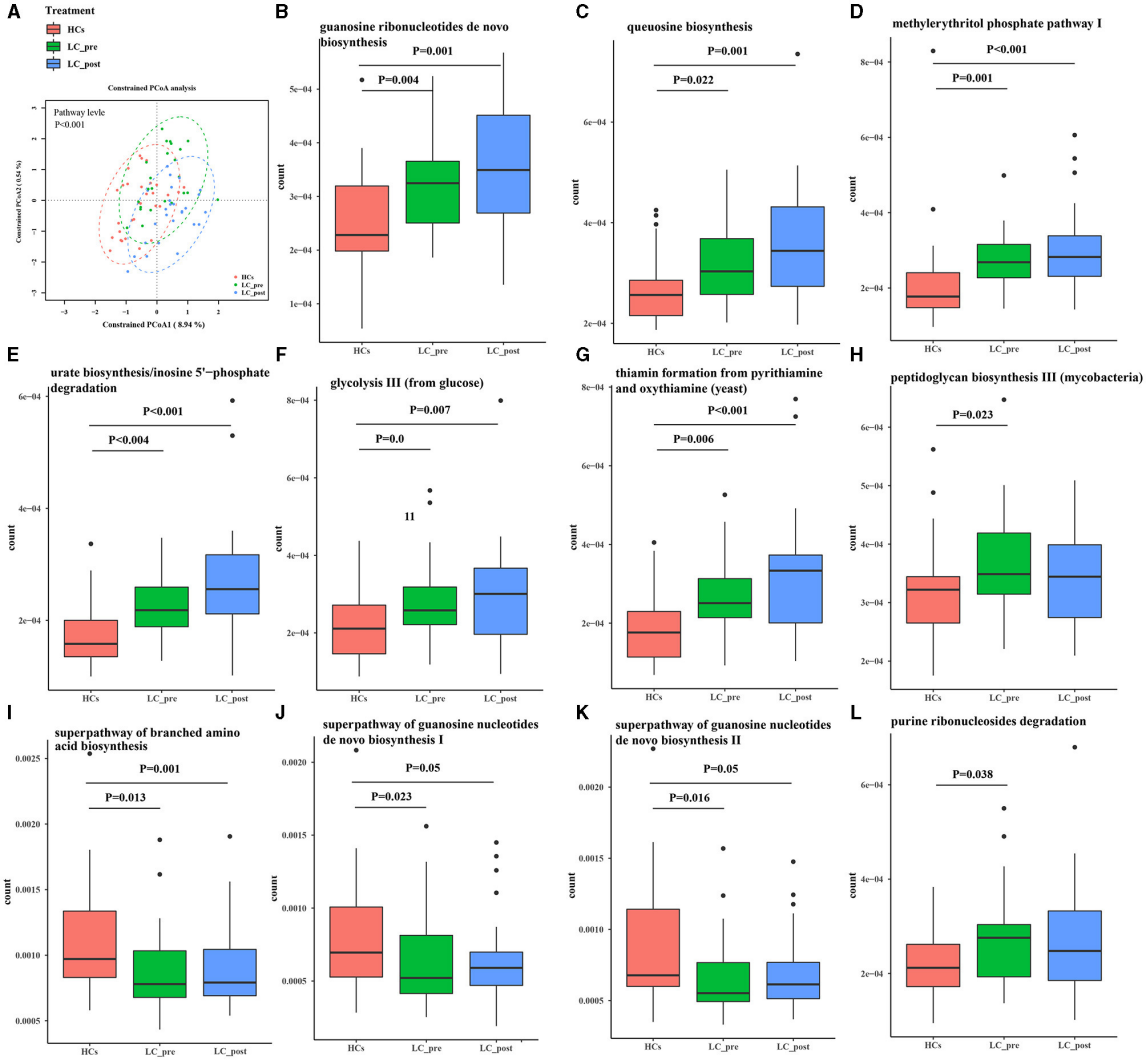
Functional pathways of the gut microbiome in cirrhotic patients before (LC-pre, green) and after (LC-post, blue) lactitol intervention and healthy controls (HCs, red). The pathways were annotated by HUMAnN2. **(A)** Beta diversity analyses of the Bray–Curtis similarity index scores of microbial pathways between the three groups, and statistical significance was measured using adonis analysis. Ellipses show 95% confidence intervals, and the different colors of ellipses represent different groups. **(B–L)** Wilcox test of the top 50 microbial metabolic pathways between groups. Data were shown as box plots with the median and 25th to 75th percentiles. Benjamini–Hochberg correction was further applied to adjust derived *p*-values. Only pathways with p-values under a threshold of 0.05 were considered as significant.

**Figure 3:**
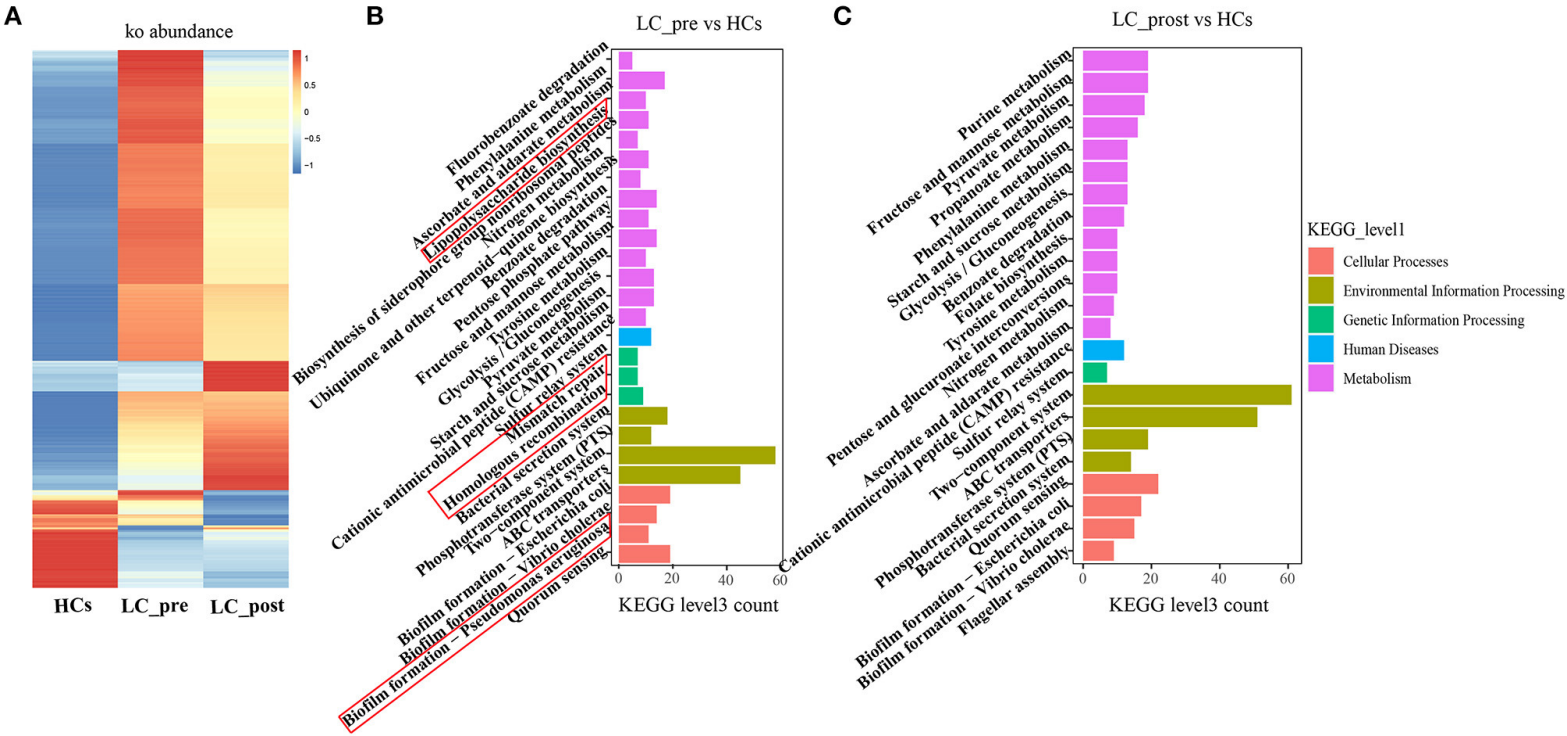
KEGG pathway enrichment analyses. **(A)** Heatmap analysis shows the difference among the cirrhotic patients before (LC-pre) and after (LC-post) lactitol intervention and healthy controls (HCs). **(B)** the number of differential KEGG metabolism level 3 from LC-pre vs. HCs. **(C)** Abundance of differential KEGG metabolism level 3 from LC-post vs. HCs. KEGG, Kyoto Encyclopedia of Genes and Genomes.

Metagenomic analysis revealed a total of 469 functional metabolic pathways from all fecal samples. A scatter plot based on principal coordinate analysis obtained from the Bray–Curtis distance matrix of functional metabolic pathways showed significant differences among LC-pre, LC-post, and HC groups (*P* < 0.001; Figure 2A). Subsequently, the differences in the top 50 abundant functional metabolic pathways were tested between LC-pre and HC groups and between LC-post and HC groups by the Wilcox test. Among the significantly differentially abundant biological processes between LC-pre and HC groups (*P* < 0.05), six pathways were upregulated (including guanosine ribonucleotide *de novo* biosynthesis, queuosine biosynthesis, methylerythritol phosphate pathway I, urate biosynthesis/inosine 5′-phosphate degradation, glycolysis III, and thiamin formation from pyrithiamine and oxythiamine) (Figures 2B–G), two showed minimal alterations (peptidoglycan biosynthesis III and superpathway of branched amino acid biosynthesis) (Figures 2H,I), and the alterations in the rest were similar to the levels observed in HCs (including superpathway of guanosine nucleotides de novo biosynthesis I, superpathway of guanosine nucleotides de novo biosynthesis II and purine ribonucleoside degradation after 4-week lactitol intervention) (Figures 2J–L). the differential abundance of the dominant KEGG Orthologs (KOs) among LC-post, LC-pre and HCs was observed using Heatmap of KOs analysis (Figure 3A). Further characterization of the functionalities of KOs in metagenomes of patients pre- actitol treatment by comparison with HCs, of note is that LPS biosynthesis, homologous recombination and mismatch repair (vital for horizontal transfer of pathogenic genes), and Pseudomonas aeruginosa biofilm formation (vital for translocation infection) were enriched in metagenomes of LC-pre (Figure 3B, Supplementary Material 2A), which is different from LC-post vs. HCs (Figure 3C, Supplementary Material 2B). Additionally, we also assigned the significant detection gene to the Cluster of Orthologous Groups (COG) database for microbialfunction analysis, and distribution and differences in relative abundances of COG categories were showed in Supplementary Figures 3A–R. Importantly, the count of COG categories of Defense mechanisms were increased significantly in LC-post when compared with LC-pre (Supplementary Figure 3I). We also found that the differentially expressed carbohydrate-active enzymes (CAZy) between the three groups mainly included glycoside hydrolases (GHs), glycosyltransferases (GTs), polysaccharide lyases (PLs), carbohydrate esterases (CEs), and carbohydrate-binding modules (CBMs) (Supplementary Figures 4A–C, Foldchange > 2 and *P* < 0.05 after adjusting for the Bonferroni correction).

**Figure 4:**
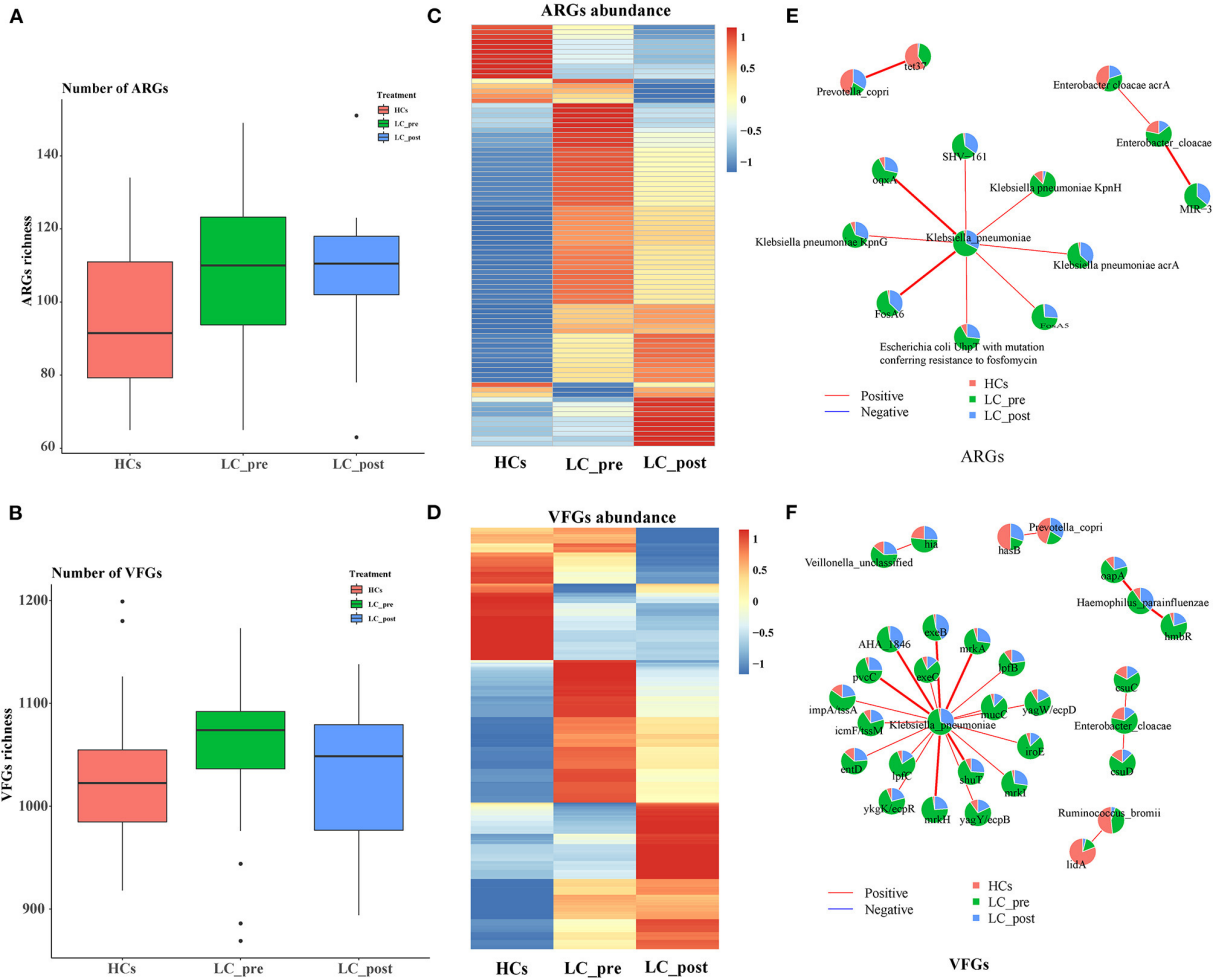
Comparison of fecal ARGs and VFGs in subjects of cirrhotic patients before (LC-pre, green) and after (LC-post, blue) lactitol intervention and healthy controls (HCs, red), and correlation analysis between discriminatory genes and bacteria. **(A)** comparison of ARGs enrichment between the three groups. **(B)** Comparison of VFG enrichment between the three groups. **(C)** Heatmap of differential ARGs. **(D)** Heatmap of differential VFGs. **(E)** Correlation between ARGs and bacteria. **(F)** Correlation between VFGs and bacteria. Wilcoxon rank-sum test with a significance level of *P* < 0.05. ARGs, antibiotics resistance genes; VFGs, virulence factor genes.

We also profiled particular genes related to antibiotic resistance and known pathogenic factors in the gut microbiome data by comparison with CARD, and VFDB databases, and 218 ARGs, and 1560 VFGs were derived (Supplementary Materials 3A, B). In terms of ARGs, matching against the CARD database revealed a significantly higher number of ARGs in both LC-pre and LC-post groups than the HC group (Figure 4A). In contrast, mapping VFGs against the VFDB database showed a significant increase in the number of VFGs in patients compared with HCs, but the number of VFGs tended to be reduced in the LC-post group and was similar to that in the HCs (Figure 4B). Specifically, compared with HCs, patients in the LC-pre group were characterized by 32 (20% of total 160 ARGs) cirrhosis-enriched ARGs, 119 (7.6% of total 1560 VFGs) cirrhosis-enriched VFGs, 4 (2.5% of total 160 ARGs) cirrhosis-depleted ARGs, and 42 (2.7% of total 1560 VFGs) cirrhosis-depleted VFGs (Figure 4C, Fold change > 2 and adjusted *P* < 0.01). After 2 months of lactitol supplementation, we found 7 (4.3% of total 160 ARGs) lactitol-upregulated ARGs, 44 (2.8% of total 1560 VFGs) lactitol-upregulated VFGs, 15 (9.37% of total 160 ARGs) lactitol-downregulated ARGs, and 80 (5.13% of total 1560 VFGs) lactitol-downregulated VFGs in the microbiome of the LC-post group (Figure 4D, Fold change > 2 and adjusted *P* < 0.01). Among these lactitol-susceptibility ARGs, we found that 22% of ARGs belonged to *Klebsiella*, 19.6% to *Escherichia*, 16% to *Enterobacteriaceae*, and 8.3% to *Enterobacter*. Additionally, 17.3% of VFGs belonged to *Klebsiella*, 12.4% to *Escherichia*, 8.1% to *Salmonella*, and 5.9% to *Pseudomonas*. Network analysis between ARGs and bacterial abundances mainly generated three different covarying clusters: (1) *P. copri* (enriched in HCs) significantly positively correlated with tet37, (2) *En. cloacae* (enriched in LC-post) with acrA and MIR-3, and 3) *K. pneumonia* (enriched in LC-pre) with SHV-161, KpnH, oqxA, KpnG, acrA, FosA6, UhpT, and FosA5 (Figure 4E, *P* < 0.05). Additionally, network analysis between VFGs and bacterial abundances mainly generated six different covarying clusters, especially for the *K. pneumonia* (enriched in LC-pre) cluster, which was significantly positively associated with 16 virulence factors, such as AHA-1846, exeB, mrkA, and lefB, vagw/ecpD (Figure 4F, *P* < 0.05). Importantly, these ARGs and VFGs that were correlated to *K. pneumonia* were significantly decreased after the 4-week lactitol intervention.

### Altered Fecal Metabolites After Lactitol Consumption

**Figure 5:**
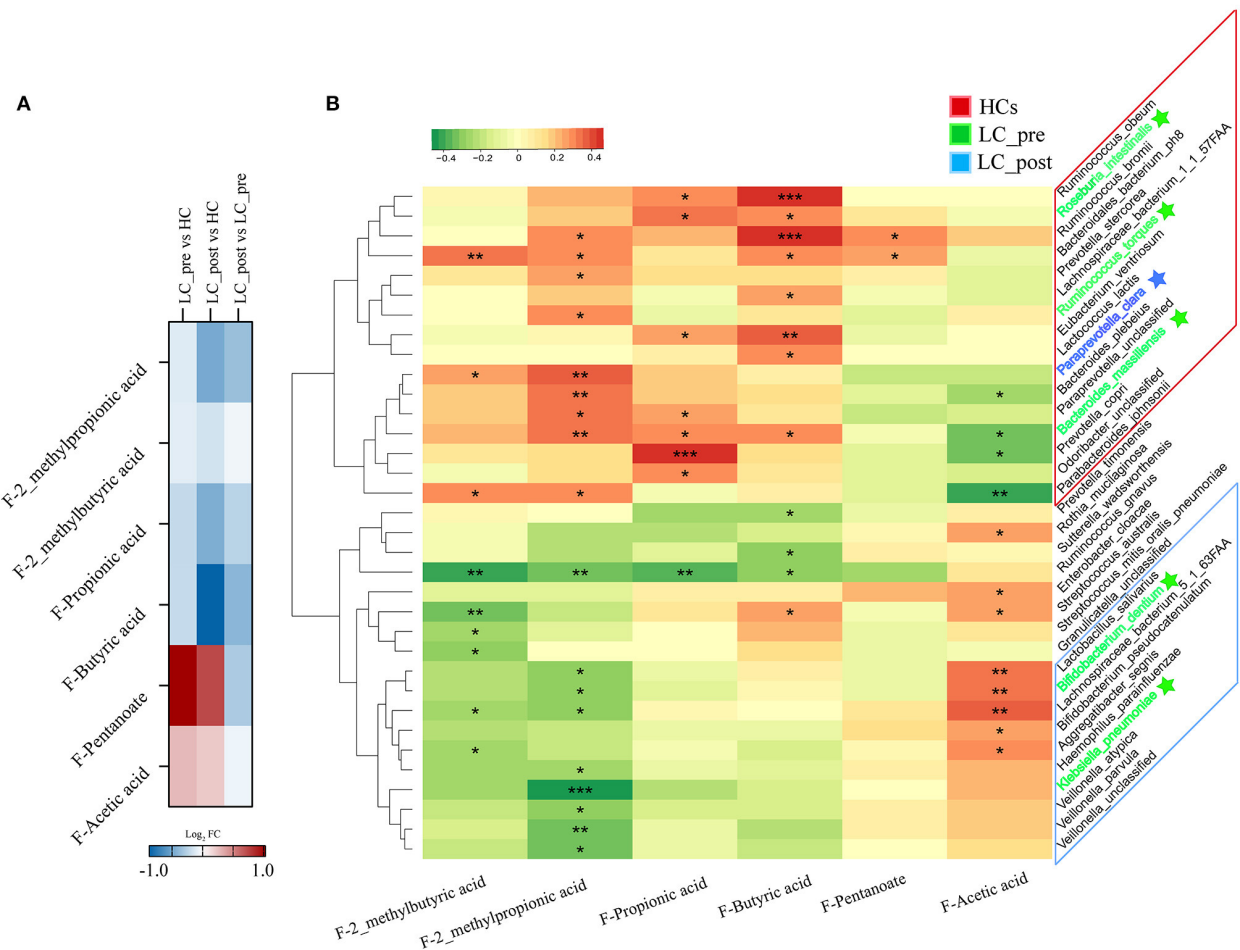
Effect of lactitol on fecal SCFAs. **(A)** Heatmap of fecal SCFAs. **(B)** Correlation between fecal SCFAs and discriminatory species. SCFAs, short chain fatty acids; LC-pre (green), cirrhotic patients before lactitol intervention; LC-post (blue), cirrhotic patients before after lactitol intervention; HCs (red), healthy controls. *p < 0.05, **p < 0.01, ***p < 0.001.

To investigate the functional consequences and causes of microbiome shifts, we further performed metabolome analysis of SCFAs and BAs in fecal samples and BAs in serum. The overall metabolic signatures of LC-post patients were significantly different from those of HCs but similar to those of LC-pre patients (Supplementary Figure 5, *P* < 0.01). The fecal SCFA profiles of the LC-pre group displayed increases in pentanoic and acetic acids and decreases in butyric, propionic, 2-methylpropionic, and 2_methylbutyric acids compared with the HCs (Figure 5A). Lactitol treatment further reduced 2_methylpropionic, propionic acid, and butyric acid and fecal pentanoate but had a minimal effect on 2_methybutyric and acetic acid. Overall, co-occurrence analysis showed that discriminatory bacterial species were strongly correlated with the fecal SCFAs (Figure 5B). Within the fecal SCFA and bacteria correlation profile, differential bacterial species mainly generated two different covarying clusters: (1) a cluster of 2-methylpropionic acid, propionic acid, and butyric acid significantly positively associated with bacteria mainly enriched in HCs (except four species: one enriched in LC-post and three in LC-pre) and (2) acetic acid with species enriched in the LC-post group (except for two species enriched in group LC-pre) (32).

**Figure 6:**
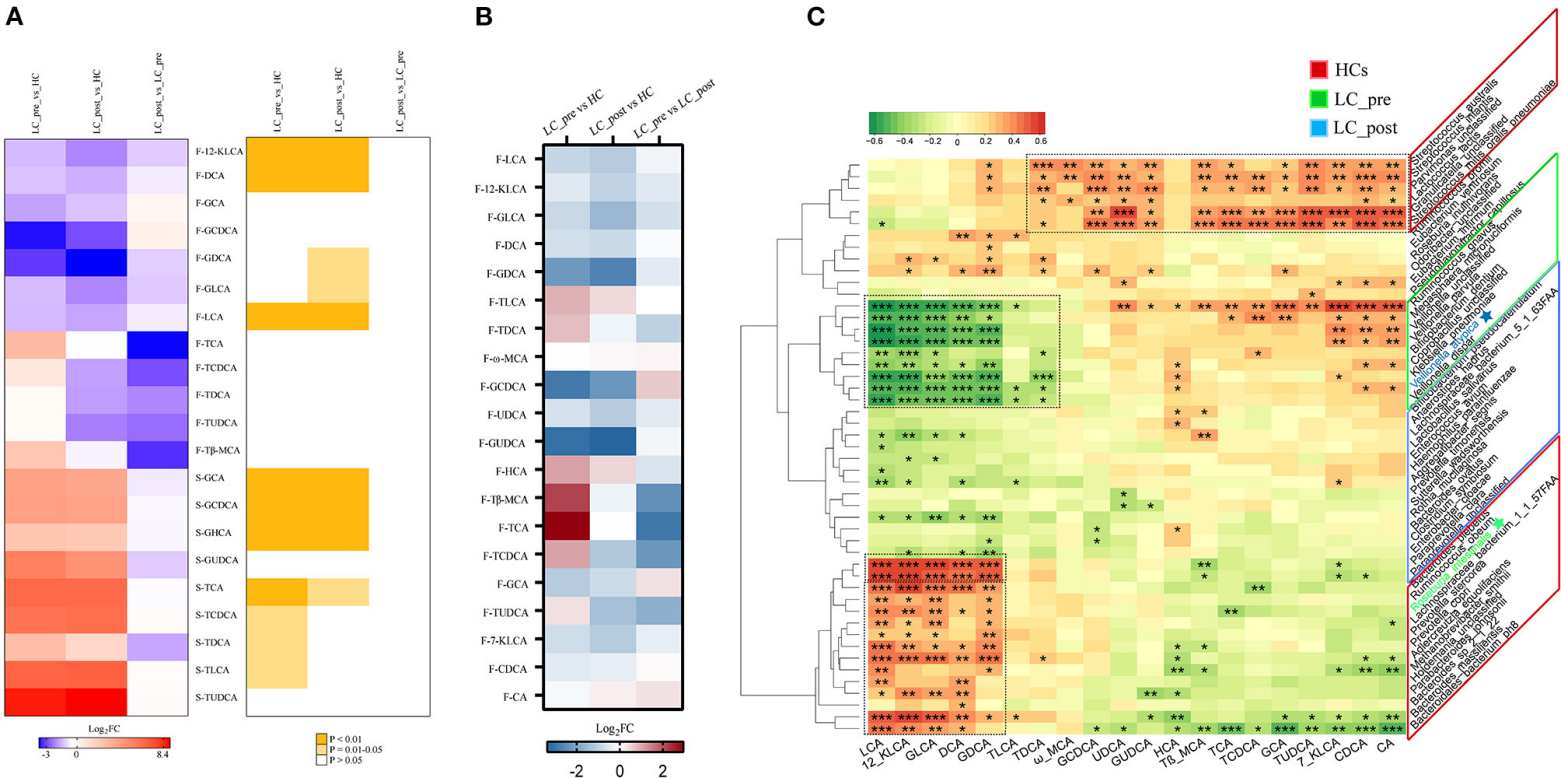
Effect of lactitol on fecal and serum BAs. **(A)** Discriminatory BA levels (Foldchange > 2) and associated *P* values for LC-pre vs. HC, LC-post vs. HCs, and LC-pre vs. LC-post. **(B)** Heatmap of fecal BAs. **(C)** Correlation between fecal BAs and discriminatory species. BAs, bile acids; LC-pre (green), cirrhotic patients before lactitol intervention; LC-post (blue), cirrhotic patients before after lactitol intervention; HCs (red), healthy controls. Benjamini–Hochberg correction was further applied to adjust derived *p*-values. Only pathways with *p*-values under a threshold of 0.05 were considered as significant. *p < 0.05, **p < 0.01, ***p < 0.001.

We also observed similar trends for both discriminatory serum and fecal BA profiles in LC-pre vs. HC groups and LC-post vs. HC groups but different trends for the most discriminatory BAs in fecal and serum samples in cirrhotic patients compared with HCs (Figure 6A). Lactitol intervention reduced some BAs, especially F-TCA, F-TβMCA, and F-TCDCA (Supplementary Material 4, VIP>1 and log2FD>1 or < -1.), but without statistical significance (*P* > 0.05). We also explored the potential correlations between discriminatory bacterial species and fecal BAs among the three groups. No significant positive associations were observed between three slightly increased fecal BAs, including F-GCDCA, F-GCA, and F-CA, in lactitol intervention groups and discriminatory species enriched in the LC-post group (Figures 6B,C). Additionally, F-LCA, F-12_KLCA, F-GLCA, F-DCA, and F-GDCA were positively significantly associated with two species (belonging to *Paraprevotella*) enriched in the LC-post group and 11 species (mainly belonging to *Bacteroides* and *Prevotella*) enriched in HCs and negatively significantly associated with eight species (mainly belonging to *Veillonella*) enriched in the LC-pre group. We also found that F-CA, F-CDCA, F-UDCA, and their conjugated BAs were positively significantly correlated with five species (three belonging to *Streptococcus*, one to *Parvimonas*, and one to *Granulicatella*) enriched in HCs (Figure 6C).

## Discussion

In this study, we performed the analysis of intestinal microbiome and metabolome with respect to lactitol-related supplement in liver cirrhotic patients. We identified species-level bacteria and metabolites, such as SCFAs and BAs, that were associated with lactitol administration in cirrhotic patients. Although no significant differences were found in microbial diversity among the groups, significant differences in the functional genes of the intestinal microbiome were found, suggesting that the effects of lactitol on the intestinal microbiome presented as alterations in the functionality of the microbiome, rather than changes in the diversity of microbiota. Particularly, lactitol treatment resulted in a significant shift of *K. pneumoniae* at the strain level as observed that 40% of patients shift from cirrhotic patient-enriched strain clusters to HC-enriched strain clusters.

Dysbiosis in gut microbiome composition is reported to correlate with cirrhosis severity (33), particularly associated with systemic inflammation and bacterial translocation (34). The changes observed in opportunistic pathogens in cirrhotic patients and HCs involve different strain *K. pneumonia*, which has been reported as cirrhosis associated species in previous studies (33, 35). We additionally found species enriched in cirrhotic patients, including *Eu. rectale, R. intestinalis, R. hominis, V. atypical, R. gnavus*, and *L. salivarius*, which were consistent with previous research (35). Our data then showed that 4 weeks of lactitol supplementation lead to decrease of cirrhosis-enriched species including pathogenic *K. pneumonia* (36) and *V. dispar* (37, 38), and increase of some health-beneficial species including *B. longum, B. pseudocatenulatum, L. salivarius* and *B. ovatus* (39). Previous studies have also demonstrated that lactitol increased the abundance of the first two species in the gut (11). Of particular note, *B. dentium*, an opportunistic pathogen enriched in cirrhotic patients (35), was also found to be enriched in the patients in this study, and reduced in abundance after lactitol supplementation.

We discovered that the lactitol supplement-modulated pathways include pathways associated with metabolic and liver function improvements, such as increased folate biosynthesis, propanoate metabolism, purine metabolism, pentose/glucuronate interconversions etc., and decreased lipopolysaccharide (LPS) and biofilm, endotoxin biosynthesis. Particularly, the alterations of functional pathways after 2-month lactitol supplement became more similar to that of HC. Therefore, we conclude that lactitol intervention could improve outcomes in cirrhosis-related intestinal microbiome dysbiosis, extending the observations from the previous study (40). Shifts in the abundance of these bacteria were correlated with alterations in functional pathways and some pathogenic genes, including ARGs and VFGs. For example, eight key ARGs and 18 key VFGs was identified to be associated with *K. pneumonia*, and decreased significantly after lactitol sumplemention. We postulate that the strain-level shift in *K. pneumoniae* contributed to these marked alterations in ARGs and VFGs as a result of lactitol treatment. Intestinal VFGs were suggested to influence host immune homeostasis (41) and be associated with disease severity (42). Of note, we found that alterations in the composition of lactitol-treated VFGs were correlated with the strain-level changes in *K.pneumonia*, which accounted for shifts in the highest proportion of differentiated VFGs in the gut microbiome of patients in LC-pre vs. LC-post groups. Lower VFG levels in *K. pneumonia* suggested weak invasiveness and reduced pathogenicity of the pathogen in lactitol-treated patients. Considering the probiotic properties of *B. longum* and *B. pseudocatenulatum*, a high abundance of both species in the LC-post group could also indicate an improvement in gut dysbiosis and therapeutic outcomes for hepatic cirrhosis.

SCFAs, particularly butyrate, have roles in the stimulation of tight junction and mucous production (43), reduction in systemic inflammation (44), and mediation of intestinal hormones (45). Although lactitol promoted the growth of *Bifidobacteria*, we did not find increase of fecal SCFAs in liver cirrhotic patients, which was reported in lactitol-treated healthy adults in the study by Finney and his colleagues (46). In fact, lactitol treatment further decreased the concentrations of intestinal SCFAs, except for 2-methylbutyric acid and acetate with no significant changes. The phenomena can potentially be explained by the fact that most species positively related to fecal 2-methylbutyric acid, methylpropionic acid, propionic acid, and butyric acid were enriched in the microbiome of HCs. Cirrhosis is always accompanied by a decreased conversion of primary to secondary fecal BAs (47). BA metabolism was found to be associated with BA biosynthesis in the liver and biotransformation by intestinal microbiota (48). Our results indicated that most of discriminatory secondary BAs in feces tended to be decreased in the LC-pre and LC-post groups compared with HCs. In contrast, the alterations in discriminatory serum BAs showed opposite trends in both patient groups vs. HCs. Together, these results suggested the presence of hepatocytosis and enterohepatic circulatory deficiency in cirrhotic patients and were consistent with the higher total serum BA levels in both patient groups compared with HCs measured by clinical biochemical tests. Higher BA (including DCA, LCA, CDCA, and TCDCA) exposure may lead to cytotoxicity (49) and have cancer-promoting effects (50). However, both the clinical biochemical tests and BA mass spectrometry results showed that lactitol treatment had a minimal influence on serum BAs. Interestingly, lactitol treatment clearly reduced fecal BAs, especially some taurine conjugates, including F-TCA, F-TDCA, F-TCDCA, F-TUDCA, and F-TβMCA, although without statistical significance. Other previous studies of probiotic supplementation in humans also reported no metabolic changes between pre- and post-probiotic intervention (51). There is undoubtedly a correlation between intestinal microbiome dysbiosis and metabolic dysregulations (52). The abundance and diversity of bacteria that perform the various BA transformations vary widely. We found that bacteria enriched in HCs were classified into two clusters according to their relationships with fecal BAs: 13 species for LCA, KLCA, GLCA, DCA, and GDCA and 6 for the rest of the fecal BAs. However, those enriched in the LC-post group did not show clear or significant correlations with fecal BAs, except for two species belonging to *Paraprevotella*. Microbiota alterations affect host signaling but not necessarily BA synthesis (53). The phenomenon can be explained to a certain extent by the minor fluctuations in fecal BAs after a 4-week lactitol intervention. BA homeostasis in the circulation pool is necessary for normal health (48). For example, LCA and DCA are takeda G protein-coupled receptor (TGR5) agonists, and TGR5 activation in colonic L cells is involved in several metabolic activities, including energy homeostasis, thermogenesis, insulin signaling inflammation, and BA uptake (54). Restoration of the intestinal BA pool increases colonic RORγ+ regulatory T cell levels and ameliorates host susceptibility to inflammation (55). In the current study, the impact of lactitol on the microbiome was evident, and the lactitol-induced minor fluctuations in BAs theoretically influenced the course of cirrhotic disease states. Whether the effects of these changes on the disease are beneficial deserves further follow-up investigations to monitor disease progression with a larger number of patients and a multi-center cohort.

To our knowledge, this is the first study to combine microbiome with targeted metabolome analyses to uncover the role of lactitol supplementation in regulating the intestinal microbiome and metabolic dysbiosis in cirrhotic patients, and our findings extended the results of previous clinical trials for lactitol. Significant alterations were found in the microbiome profiles, including bacterial communities, microbiota-associated pathways, ABGs, and VFGs, in patients pre- and post-lactitol treatment, but we only identified minor lactitol-induced changes in BA and SCFA metabolites. However, one limitation of the current study was that it is only a single-center study performed in a small cohort. Therefore, it is necessary to perform future large-scale, multicenter, randomized, and placebo-controlled trials and experiments with a lactitol-treated cirrhotic rat model to further validate the lactitol-induced alterations are associated with improvements in disease status and the prevention of cirrhosis progression.

## Conclusion

The results of the present study indicated that supplement of lactitol was associated with changes in bacterial abundances at the strain level were correlated with altered functions of the microbiome. Our results extended the observations from previous clinical trials of lactitol by providing evidence on lactitol-induced improvements in the microbiome of hepatic cirrhotic patients based on fecal metabolic pathways, ARG and VFG pools. Given the increasing importance of microbial resistance to antibiotics, fecal ARGs and VFGs may become the next research topic of high interest in probiotic or prebiotic clinical trials because epigenetic signatures have not yet been shown to predict the therapeutic results of microecological modulators. Lactitol-induced improvements in clinical outcomes require further follow-up investigations on to verify the clinical relevance of microbiome alterations.

## Data Availability Statement

The raw sequencing data have been deposited in the National Microbiology Data Center with the accession number is NMDC10017859. Further information and requests for resources and reagents should be directed to and will be fulfilled by the corresponding author, Zhongwen Wu (wuzhongwen@zju.edu.cn).

## Ethics Statement

The studies involving human participants were reviewed and approved by the Ethics Committee of the First Affiliated Hospital, School of Medicine, Zhejiang University (reference number: 2017HL668-1). The patients/participants provided their written informed consent to participate in this study.

## Author Contributions

ZW and LL designed the study. HL, XP, YY, HZ, XZ, XL, CZ, LL, and ZW enrolled the subjects, collected clinical samples, and performed the experiments. LC, HL, ZW, and JW performed the analysis. HL and JW wrote the manuscript. All authors contributed to the article and approved the submitted version.

## Funding

This work was supported by the National Key Research and Development Program of China (2018YFC2000500), National Natural Science Foundation of China (81874038), the Project of Zhejiang Provincial Natural Science Foundation (LQ17H030002, LQ20H030010 and LGF19H030009), and the Opening Foundation of the State Key Laboratory for Diagnosis and Treatment of Infectious Diseases and Collaborative Innovation Center for Diagnosis and Treatment of Infectious Diseases, the First Affiliated Hospital, College of Medicine, Zhejiang University (SKLID2019KF03).

## Conflict of Interest

The authors declare that the research was conducted in the absence of any commercial or financial relationships that could be construed as a potential conflict of interest.

## Publisher's Note

All claims expressed in this article are solely those of the authors and do not necessarily represent those of their affiliated organizations, or those of the publisher, the editors and the reviewers. Any product that may be evaluated in this article, or claim that may be made by its manufacturer, is not guaranteed or endorsed by the publisher.

## References

[1] Lin J Wu JF Zhang Q Zhang HW Cao GW. Virus-related liver cirrhosis: molecular basis and therapeutic options. World J Gastroenterol. (2014) 20:6457–69. 10.3748/wjg.v20.i21.6457 24914367 PMC4047331

[2] Trebicka J Bork P Krag A Arumugam M. Utilizing the gut microbiome in decompensated cirrhosis and acute-on-chronic liver failure. Nat Rev Gastroenterol Hepatol. (2021) 18:167–80. 10.1038/s41575-020-00376-3 33257833

[3] Szabo G Bala S Petrasek J Gattu A. Gut-liver axis and sensing microbes. Dig Dis. (2010) 28:737–44. 10.1159/000324281 21525758 PMC3211517

[4] Abu-Shanab A Quigley EM. The role of the gut microbiota in nonalcoholic fatty liver disease. Nat Rev Gastroenterol Hepatol. (2010) 7:691–701. 10.1038/nrgastro.2010.172 21045794

[5] Quigley EM Marsh MN Shaffer JL Markin RS. Hepatobiliary complications of total parenteral nutrition. Gastroenterology. (1993) 104:286–301. 10.1016/0016-5085(93)90864-9 8419252

[6] Terjung B Spengler U. Atypical p-ANCA in PSC and AIH: a hint toward a “leaky gut”? Clin Rev Allergy Immunol. (2009) 36:40–51. 10.1007/s12016-008-8088-8 18626795

[7] Usami M Miyoshi M Yamashita H. Gut microbiota and host metabolism in liver cirrhosis. World J Gastroenterol. (2015) 21:11597–608. 10.3748/wjg.v21.i41.11597 26556989 PMC4631963

[8] Lee NY Suk KT. The role of the gut microbiome in liver cirrhosis treatment. Int J Mol Sci. (2020) 22. 10.3390/ijms22010199 33379148 PMC7796381

[9] Sung CM Lin YF Chen KF Ke HM Huang HY Gong YN. Predicting clinical outcomes of cirrhosis patients with hepatic encephalopathy from the fecal microbiome. Cell Mol Gastroenterol Hepatol. (2019) 8:301–18 e2. 10.1016/j.jcmgh.2019.04.008 31004827 PMC6718362

[10] H. Fukui. Gut Microbiome-based Therapeutics in Liver Cirrhosis: Basic Consideration for the Next Step. J Clin Transl Hepatol. (2017) 5:249–60. 28936406 10.14218/JCTH.2017.00008 PMC5606971

[11] Li XQ Zhang XM Wu X Lan Y Xu L Meng XC. Beneficial effects of lactitol on the composition of gut microbiota in constipated patients. J Dig Dis. (2020) 21:445–53. 10.1111/1751-2980.12912 32483935

[12] Gluud LL Vilstrup H Morgan MY. Non-absorbable disaccharides vs. placebo/no intervention and lactulose vs. lactitol for the prevention and treatment of hepatic encephalopathy in people with cirrhosis. Cochrane Database Syst Rev. (2016) 4:CD003044. 10.1002/14651858.CD003044.pub3 27089005

[13] Chen C Yu X Lu H Xiao D Mao W Li L. Antioxidant protective effects of lactitol against endotoxemia in patients with chronic viral hepatitis. Mol Med Rep. (2013) 7:401–5. 10.3892/mmr.2012.1188 23165913

[14] Maydeo. Lactitol or lactulose in the treatment of chronic constipation: result of a systematic. J Indian Med Assoc. (2010) 108:789–92. 21510584

[15] Chen C Li L Wu Z Chen H Fu S. Effects of lactitol on intestinal microflora and plasma endotoxin in patients with chronic viral hepatitis. J Infect. (2007) 54:98–102. 10.1016/j.jinf.2005.11.013 17049992

[16] Bolger AM Lohse M Usadel B. Trimmomatic: a flexible trimmer for Illumina sequence data. Bioinformatics. (2014) 30:2114–20. 10.1093/bioinformatics/btu170 24695404 PMC4103590

[17] Langmead B Salzberg SL. Fast gapped-read alignment with Bowtie 2. Nat Methods. (2012) 9:357–9. 10.1038/nmeth.1923 22388286 PMC3322381

[18] Segata N Waldron L Ballarini A Narasimhan V Jousson O Huttenhower C. Metagenomic microbial community profiling using unique clade-specific marker genes. Nat Methods. (2012) 9:811–4. 10.1038/nmeth.2066 22688413 PMC3443552

[19] Franzosa EA McIver LJ Rahnavard G Thompson LR Schirmer M Weingart G. Species-level functional profiling of metagenomes and metatranscriptomes. Nature methods. (2018) 15:962–8. 10.1038/s41592-018-0176-y 30377376 PMC6235447

[20] Segata N Izard J Waldron L Gevers D Miropolsky L Garrett WS. Metagenomic biomarker discovery and explanation. Genome biology. (2011) 12:R60. 10.1186/gb-2011-12-6-r60 21702898 PMC3218848

[21] Li D Liu CM Luo R Sadakane K Lam TW. MEGAHIT: an ultra-fast single-node solution for large and complex metagenomics assembly via succinct de Bruijn graph. Bioinformatics. (2015) 31:1674–6. 10.1093/bioinformatics/btv033 25609793

[22] T. Seemann. Prokka: rapid prokaryotic genome annotation. Bioinformatics. (2014) 30:2068–9. 10.1093/bioinformatics/btu153 24642063

[23] Fu L Niu B Zhu Z Wu S Li W. CD-HIT: accelerated for clustering the next-generation sequencing data. Bioinformatics. (2012) 28:3150–2. 10.1093/bioinformatics/bts565 23060610 PMC3516142

[24] Patro R Duggal G Love MI Irizarry RA Kingsford C. Salmon provides fast and bias-aware quantification of transcript expression. Nat Methods. (2017) 14:417–9. 10.1038/nmeth.4197 28263959 PMC5600148

[25] uerta-Cepas J Szklarczyk D Forslund K Cook H Heller D Walter MC. eggNOG 4.5: a hierarchical orthology framework with improved functional annotations for eukaryotic, prokaryotic and viral sequences. Nucleic Acids Res. (2016) 44:D286-93. 10.1093/nar/gkv1248 26582926 PMC4702882

[26] Petersen M Meusemann K Donath A Dowling D Liu S Peters RS. Orthograph: a versatile tool for mapping coding nucleotide sequences to clusters of orthologous genes. BMC Bioinformatics. (2017) 18:111. 10.1186/s12859-017-1529-8 28209129 PMC5312442

[27] Alcock BP Raphenya AR Lau TTY Tsang KK Bouchard M Edalatmand A. CARD 2020: antibiotic resistome surveillance with the comprehensive antibiotic resistance database. Nucleic Acids Res. (2020) 48:D517-D525. 10.1093/nar/gkz935 31665441 PMC7145624

[28] Liu B Zheng D Jin Q Chen L Yang J. VFDB 2019: a comparative pathogenomic platform with an interactive web interface. Nucleic Acids Res. (2019) 47:D687–92. 10.1093/nar/gky1080 30395255 PMC6324032

[29] Buchfink B Xie C Huson DH. Fast and sensitive protein alignment using DIAMOND. Nat Methods. (2015) 12:59–60. 10.1038/nmeth.3176 25402007

[30] Wang S Yang L Hu H Lv L Ji Z Zhao Y. Characteristic gut microbiota and metabolic changes in patients with pulmonary tuberculosis. Microb Biotechnol. (2021). 10.1111/1751-7915.13761 33599402 PMC8719804

[31] Jari OksanenFGB Friendly M Kindt R Legendre P McGlinn D Minchin PR. vegan: Community Ecology Package. R package version 2.5-7. Avaialble online at: https://CRAN.R-project.org/package=vegan (2020).

[32] Nonalcoholic Fatty Liver Disease. Am Fam Physician. (2020) 102. 33179900

[33] Sole C Guilly S Da Silva K Llopis M Le-Chatelier E Huelin P. Alterations in Gut Microbiome in Cirrhosis as Assessed by Quantitative Metagenomics: Relationship With Acute-on-Chronic Liver Failure and Prognosis. Gastroenterology. (2021) 160:206–18 e13. 10.1053/j.gastro.2020.08.054 32941879

[34] Giannelli V Gregorio VDi Iebba V Giusto M Schippa S Merli M. Microbiota and the gut-liver axis: bacterial translocation, inflammation and infection in cirrhosis *World J Gastroenterol*. (2014) 20:16795–810. 10.3748/wjg.v20.i45.16795 25492994 PMC4258550

[35] Qin N Yang F Li A Prifti E Chen Y Shao L. Alterations of the human gut microbiome in liver cirrhosis. Nature. (2014) 513:59–64. 10.1038/nature13568 25079328

[36] Santiago A.J. Donlan R.M. Bacteriophage Infections of Biofilms of Health Care-Associated Pathogens: Klebsiella pneumoniae. EcoSal Plus. (2020) 9. 10.1128/ecosalplus.ESP-0029-2019 33118486 PMC10242522

[37] Cobo F Perez-Carrasco V Garcia-Salcedo JA Navarro-Mari JM. Bacteremia caused by Veillonella dispar in an oncological patient. Anaerobe. (2020) 66:102285. 10.1016/j.anaerobe.2020.102285 33075505 PMC7563575

[38] Loughrey AC Chew EW. Endocarditis caused by Veillonella dispar. J Infect. (1990) 21:319–21. 10.1016/0163-4453(90)94197-8 2273279

[39] Hudcovic T Kozakova H Kolinska J Stepankova R Hrncir T Tlaskalova-Hogenova H. Monocolonization with Bacteroides ovatus protects immunodeficient SCID mice from mortality in chronic intestinal inflammation caused by long-lasting dextran sodium sulfate treatment. Physiological research. (2009) 58:101–10. 10.33549/physiolres.931340 18198984

[40] Ballongue J Schumann C Quignon P. Effects of lactulose and lactitol on colonic microflora and enzymatic activity. Scand J Gastroenterol Suppl. (1997) 222:41–4. 10.1080/00365521.1997.11720716 9145445

[41] Agany DDM Pietri JE Gnimpieba EZ. Assessment of vector-host-pathogen relationships using data mining and machine learning. Comput Struct Biotechnol J. (2020) 18:1704–21. 10.1016/j.csbj.2020.06.031 32670510 PMC7340972

[42] Wang M Doenyas C Wan J Zeng S Cai C Zhou J. Virulence factor-related gut microbiota genes and immunoglobulin A levels as novel markers for machine learning-based classification of autism spectrum disorder. Comput Struct Biotechnol J. (2021) 19:545–54. 10.1016/j.csbj.2020.12.012 33510860 PMC7809157

[43] Ichikawa H Shineha R Satomi S Sakata T. Gastric or rectal instillation of short-chain fatty acids stimulates epithelial cell proliferation of small and large intestine in rats. Dig Dis Sci. (2002) 47:1141–6. 10.1023/A:1015014829605 12018914

[44] Bartolomaeus H Balogh A Yakoub M Homann S Marko L Hoges S. Short-chain fatty acid propionate protects from hypertensive cardiovascular damage. Circulation. (2019) 139:1407–21. 10.1161/CIRCULATIONAHA.118.036652 30586752 PMC6416008

[45] Zhuang M Shang W Ma Q Strappe P Zhou Z. Abundance of probiotics and butyrate-production microbiome manages constipation via short-chain fatty acids production and hormones secretion. Mol Nutr Food Res. (2019) 63:e1801187. 10.1002/mnfr.201801187 31556210

[46] Finney M Smullen J Foster HA Brokx S Storey DM. Effects of low doses of lactitol on faecal microflora, pH, short chain fatty acids and gastrointestinal symptomology. Eur J Nutr. (2007) 46:307–14. 10.1007/s00394-007-0666-7 17623227

[47] Kakiyama G Pandak WM Gillevet PM Hylemon PB Heuman DM Daita K. Modulation of the fecal bile acid profile by gut microbiota in cirrhosis. J Hepatol. (2013) 58:949–55. 10.1016/j.jhep.2013.01.003 23333527 PMC3936319

[48] Chiang JYL Ferrell JM. Bile acid metabolism in liver pathobiology. Gene Expr. (2018) 18:71–87. 10.3727/105221618X15156018385515 29325602 PMC5954621

[49] Ferslew BC Xie G Johnston CK Su M Stewart PW Jia W. Altered bile acid metabolome in patients with nonalcoholic steatohepatitis. Dig Dis Sci. (2015) 60:3318–28. 10.1007/s10620-015-3776-8 26138654 PMC4864493

[50] Bernstein H Bernstein C Payne CM Dvorak K. Bile acids as endogenous etiologic agents in gastrointestinal cancer. World J Gastroenterol. (2009) 15:3329–40. 10.3748/wjg.15.3329 19610133 PMC2712893

[51] Stadlbauer V Leber B Lemesch S Trajanoski S Bashir M Horvath A. Lactobacillus casei shirota supplementation does not restore gut microbiota composition and gut barrier in metabolic syndrome: a randomized pilot study. PLoS ONE. (2015) 10:e0141399. 10.1371/journal.pone.0141399 26509793 PMC4625062

[52] McHenry S Davidson NO. Bile acids, microbiota, and cystic fibrosis: channeling intestinal FXR signals. Cell Mol Gastroenterol Hepatol. (2020) 9:185–6. 10.1016/j.jcmgh.2019.09.004 31604089 PMC6926323

[53] Marion S Desharnais L Studer N Dong Y Notter MD Poudel S. Biogeography of microbial bile acid transformations along the murine gut. J Lipid Res. (2020) 61:1450–63. 10.1194/jlr.RA120001021 32661017 PMC7604727

[54] Wahlstrom A Sayin SI Marschall HU Backhed F. Intestinal crosstalk between bile acids and microbiota and its impact on host metabolism. Cell Metab. (2016) 24:41–50. 10.1016/j.cmet.2016.05.005 27320064

[55] Song X Sun X Oh SF Wu M Zhang Y Zheng W. Microbial bile acid metabolites modulate gut RORgamma(+) regulatory T cell homeostasis. Nature. (2020) 577:410–5. 10.1038/s41586-019-1865-0 31875848 PMC7274525

