# Supplementary material for: Lactitol Supplementation Modulates Intestinal Microbiome in Liver Cirrhotic Patients
Source: Front Med (Lausanne). 2021 Oct 14;8:762930. doi: 10.3389/fmed.2021.762930 (PMC8551616; doi:10.3389/fmed.2021.762930)
Supplement: Supplementary file 5 [file Table_5.docx]

**TABLE 1** Subject characteristics

| Characteristics | | Group HC | Group LC_pre | Group LC_post | P-value | | |
| --- | --- | --- | --- | --- | --- | --- | --- |
|  |  | N=29 (%) | N=24 (%) | | LC_pre vs HC | LC_post vs HC | LC_ post vs LC_ pre |
| Gender | Female | 8 (27.58) | 7 (29.17) | | 0.840 | 0.840 | - |
|  | Male | 21 (72.42) | 17 (70.83) | |  |  |  |
| Age | | 50.77±6.76 | 51.58±10.95 | | 0.751 | 0.751 | - |
| BMI (kg/m^2^) | | 21.58±3.05 | 22.30±2.74 | | 0.370 | 0.370 | - |
| ALT (5-40U/L) | | 25.23±10.46 | 26.83±12.23 | 31.08±18.11 | 0.678 | 0.132 | 0.297 |
| AST (8-40U/L) | | 24.97±6.52 | 34.54±14.55 | 34.83±14.98 | 0.007 | 0.005 | 0.936 |
| Albumin (35-55g/L) | | 45.34±2.71 | 41.96±4.68 | 43.24±4.85 | 0.012 | 0.196 | 0.744 |
| Globulin (20-35g/L) | | 26.27±2.98 | 30.31±5.43 | 29.98±5.15 | 0.009 | 0.012 | 0.995 |
| TB (0-21mg/dL) | | 11.37±5.29 | 25.08±14.70 | 24.87±11.82 | <0.001 | <0.001 | 1.000 |
| PLT(83-303*10^9^/L) | | 233.83±49.99 | 84.71±56.69 | 85.08±57.44 | <0.001 | <0.001 | 1.000 |
| Crea(59-104mg/dL) | | 73.50±11.96 | 69.54±11.97 | 66.42±11.31 | 0.232 | 0.034 | 0.370 |
| INR | | - | 1.18±0.14 | 1.17±0.11 | - | - | 1.000 |
| MELD | | - | 5.37±0.34 | 5.34±0.30 | - | - | 0.984 |

The data were depicted as Mean ± SD. Continuous variables with a normal distribution were assessed using two-tailed independent sample t tests, whereas the data that did not fit a normal distribution were assessed using a non-parametric Mann-Whitney test.

The MELD score formula is R= 0.378 × lnTB (mg/dL) +1.12 × lnINR +0. 957 × lnCrea (mg/dL) + 0.64(biliary or alcoholic 0, others 1).

Abbreviations: HC, Healthy controls; LC_pre, patients with cirrhosis before lactitol treatment; LC_post, those patients after lactitol treatment; BMI, body mass index; ALT, alanine aminotransferase; AST, aspartate aminotransferase; TB, total bilirubin; PLT, platelet; INR international normalized ratio; Crea, China real estate academe; MELD, model for end-stage liver disease.
