# Supplementary material for: Lactitol Supplementation Modulates Intestinal Microbiome in Liver Cirrhotic Patients
Source: Front Med (Lausanne). 2021 Oct 14;8:762930. doi: 10.3389/fmed.2021.762930 (PMC8551616; doi:10.3389/fmed.2021.762930)

**Supplementary file 1**

**Lactitol Supplementation Modulates Intestinal Microbiome in Liver Cirrhotic Patients**

Haifeng Lu^1, 2^ ^†^, Liang Chen^3^ ^†^, Xiaxia Pan^1^, Yujun Yao^1^, Hua Zhang^1, 2^, Xiaofei Zhu^1^, Xiaobin Lou^1, 2^, Chunxia Zhu^1, 2^, Jun Wang^3,4*^, Lanjuan Li^1, 2*^, Zhongwen Wu^1, 2*^

^1^, State Key Laboratory for Diagnosis and Treatment of Infectious Diseases, First Affiliated Hospital, School of Medicine, Zhejiang University, Hangzhou 310003, Zhejiang Province, People’s Republic of China

^2^, National Clinical Research Center for Infectious Diseases, First Affiliated Hospital, School of Medicine, Zhejiang University, Hangzhou 310003, Zhejiang Province, People’s Republic of China

^3^, CAS Key Laboratory of Pathogenic Microbiology and Immunology, Institute of Microbiology, Chinese Academy of Science, Beijing 100101, People’s Republic of China.

^4^, University of Chinese Academy of Sciences, Beijing 100049, People’s Republic of China.

^1^

^†^, HL and LC contributed equally.

Correspondence to Zhongwen Wu and Lanjuan Li, State Key Laboratory for Diagnosis and Treatment of Infectious Disease, the First Affiliated Hospital, School of Medicine, Zhejiang, China; wuzhongwen@zju.edu.cn, [ljli@zju.edu.cn](mailto:ljli@zju.edu.cn), and Professor Jun Wang, CAS Key Laboratory of Pathogenic Microbiology and Immunology, Institute of Microbiology, Chinese Academy of Sciences, Beijing, People’s Republic of China; [junwang@im.ac.cn](mailto:junwang@im.ac.cn).

**Patients and Methods**

**Inclusion and Exclusion Criteria for Subjects**

Briefly, we prospectively enrolled eligible subjects from July 2018 to September 2020. The inclusion criteria were as follows: (1) adults (aged from 45 to 65 years); (2) ultrasonographic findings confirming liver cirrhosis; (3) no elevated ALT (alanine transaminase) levels within the past 6 months; and (4) MELD (Model for end-stage liver disease) score and Child–Pugh Class A (≤6). Subjects who met any of the following criteria were excluded: (1) hepatitis virus infection other than HBV; (2) autoimmune hepatitis, primary biliary cholangitis, PSC, gastrointestinal cancers, hepatocellular carcinoma, or drug-induced liver injury; (3) Wilson disease or hemochromatosis; (4) excessive alcohol, cigarette, or drug consumption; (5) antibiotic use within 6 weeks; (6) underwent any surgeries (<5 years); and (7) chronic disorders associated with nutritional, metabolic, or immunological diseases. The HC group included subjects matched to the age, sex, and BMI of the LC group and were correspondingly screened and enrolled according to a routine examination within 12 weeks. HCs did not have any chronic diseases and were not taking any chronic medications, including proton pump inhibitors. The inclusion and exclusion criteria were described in our previous study [[17](#_ENREF_17)].

**Clean Read Filtering and De Novo Assembly**

Raw reads were processed to obtain high-quality clean reads according to the following standards: removed reads with ≥10% unidentified nucleotides (Ns); removed reads with > 50% bases having paired quality scores of ≤5; removed reads belonging to the human genome; and removed reads aligned to the barcode adapter. Illumina sequence data of each sample were assembled into contigs individually using MEGAHIT [MEGAHIT: an ultra-fast single-node solution for large and complex metagenomics assembly via a succinct de Bruijn graph]. By stepping over a K-mer range of 21–99, we generated sample-derived assembly. Overall, de novo assembly statistics were evaluated as a combination of percent paired or singleton reads realigning to the assembly using BWA [ab initio gene identification in metagenomic sequences]. The unmapped reads of each sample were pooled for re-assembly using MEGAHIT to generate a mixed assembly. The sample-derived assembly and mixed assembly were combined to obtain a final assembly for gene prediction and taxonomic profiling analysis. All unique open reading frames (ORF) were annotated using DIAMOND [Fast and sensitive protein alignment using DIAMOND] to the following functional databases: KEGG, CAZY, and eggNOG. After the filtering of reads, clean reads were used to generate taxonomic profiles using the k-mer-based taxonomic classifier MetaOthello [a novel data structure to support the ultra-fast taxonomic classification of metagenomic sequences with K-mer signatures]. MetaOthello applies a novel data structure named 1-Othello to support efficient querying of taxonomic information from NCBI using reads with K-mer signatures of 31 bp in length.

**Gene Prediction and Catalog Generation**

To explore the metagenomic information of the intestinal microbiota, ORFs were predicted based on the final assembly contigs (>500 bp) using MetaGeneMark. The predicted ORFs ≥300 bp from all samples were pooled, and gene pairs with greater than 95% identity (no gap allowed) and aligned reads covering over 90% of the shorter reads were grouped together using CD-HIT to reduce the number of redundant genes for the downstream assembly step. The reads were re-aligned to predict genes using BWA to count read numbers. Finally, a gene catalog was obtained from non-redundant genes with gene read counts >2.

**BA Quantification using LC-MS**

The extracts were analyzed using an LC- ESI-MS/MS system (UHPLC, ExionLCTM AD; MS, Applied Biosystems 6500 Triple Quadrupole). The analytical conditions were as follows: HPLC, Waters ACQUITY UPLC HSS T3C18 (100 mm × 2.1 mm internal diameter × 1.8 μm); Solent system, water with 0.01% acetic acid and 5 mmoL ammonium acetate (A), acetonitrile with 0.01% acetic acid (B); gradient optimized at 5% to 40% B in 0.5 min, increased to 50% B in 4 min, increased to 75% B in 3 min, increased to 75% to 95% in 2.5 min, washed with 95% B for 2 min, and switched back to 5% B (12–14 min); flow rate, 0.35 mL/min; temperature, 40℃; and injection volume, 3 μL. The effluent was alternatively connected to an ESI-triple quadrupole-linear ion trap (QTRAP)-MS. BAs detected in the current study included hyocholic acid (HCA), glycohyocholic acid (GHCA), omega-muricholic acid (ꞷ-MCA), beta-muricholic acid (β-MCA), cholic acid (CA), chenodeoxycholic acid (CDCA), tauroursodeoxycholic acid (TUDCA), glycoursodeoxycholic acid (GUDCA), taurochenodeoxycholic acid (TCDCA), taurocholic acid (TCA), ursodeoxycholic acid (UDCA), taurodeoxycholic acid (TDCA), glycocholic acid (GCA), glycolithocholic acid (GLCA), lithocholic acid (LCA), glycochenodeoxycholic acid (GCDCA), deoxycholic acid (DCA), and 3α-hydroxy-12 ketolithocholic acid (12-KLCA).

**SCFA Quantification using GC-MS.**

Stool SCFA profiles were assessed using 20 mg of stool samples with 500 μL of precooled water containing 10 μg/mL hexanoic-6,6,6-d3 acid (used as the internal standard, CAS: 55320-69-9, Sigma-Aldrich, St. Louis, MO, USA). The mixture was centrifuged at 15000 rpm for 5 min at 4℃. Then, the supernatant was filtered through a 0.22-μm Millipore filter membrane, mixed with the same volumes of ethyl acetate (5% sulfuric acid), centrifuged at 15000 rpm for 5 min at 4℃, and incubated for 30 min at 4℃. Finally, 120 μL of supernatant was added to micro-volume vial inserts with preinstalled plastic (Sigma-Aldrich) and placed in chromatography columns. The standards were added to chromatography Autosampler Vials with closures (AIJIREN®, Zhejiang, China). The quantitative and qualitative analyses were performed by GC-MS (Agilent 7890/5975C, Santa Clara, CA, USA).

Supplementary Figure 1. Effect of lactitol on fecal microbiota at the genus and species level. (A) Comparisons of fecal genus-level bacteria richness between LC-pre (green), LC-post (blue), and HC (red) groups. (B) Comparisons of fecal genus-level bacteria Shannon diversity between LC-pre (green), LC-post (blue), and HC (red) groups. (C) Comparisons of fecal species-level bacteria richness between LC-pre (green), LC-post (blue), and HC (red) groups. (D) Comparisons of fecal species-level bacteria Shannon diversity between LC-pre (green), LC-post (blue), and HC (red) groups. (E) Fecal genus-level principal coordinate analysis for LC-pre (green), LC-post (blue), and HC (red) groups. (F) Fecal species-level principal coordinate analysis for LC-pre (green), LC-post (blue), and HC (red) groups. Wilcoxon rank-sum test with a significance level of P<0.05.


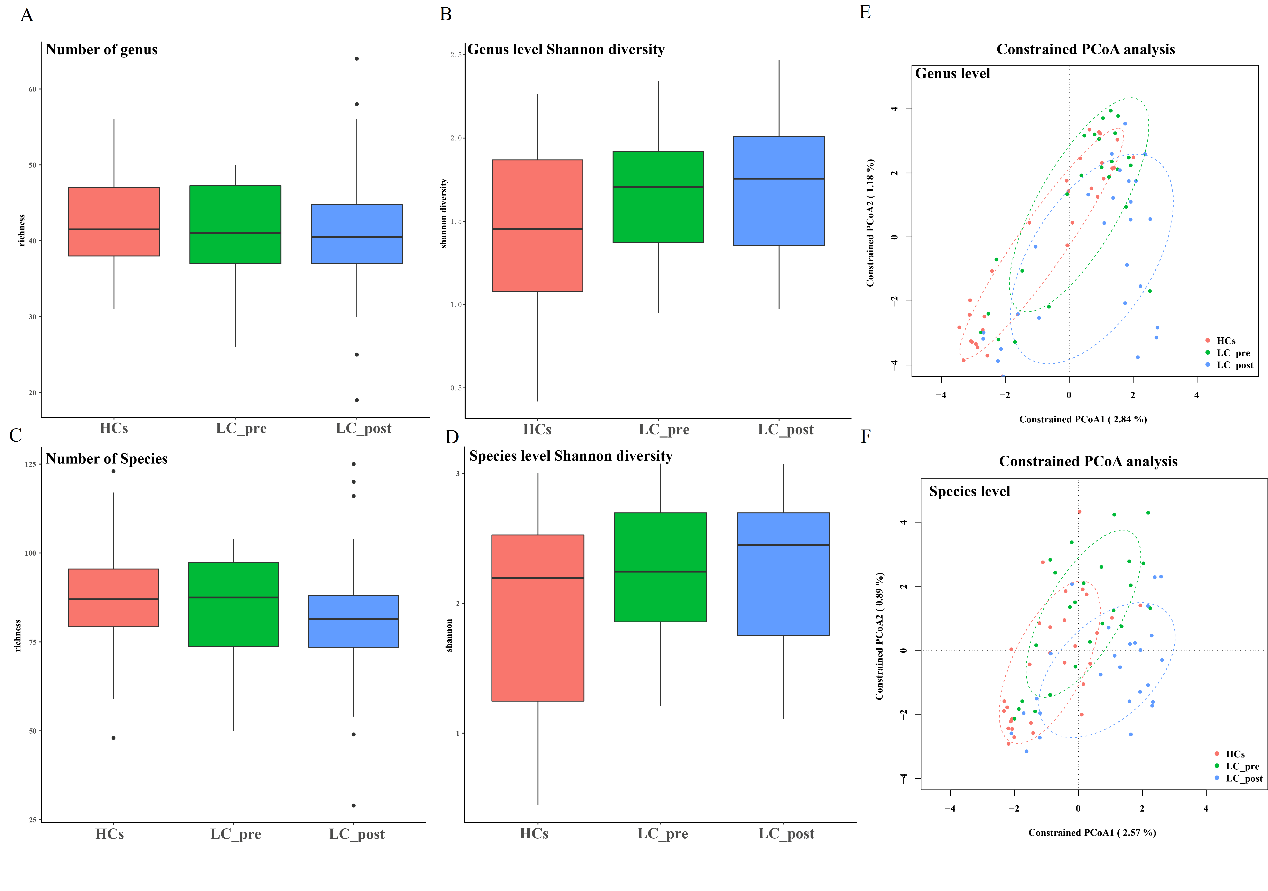


Supplementary Figure 2. Comparison of the average abundance of each bacterial genus (A) and species (B) in group LC_pre, LC_post and HCs, respectively.


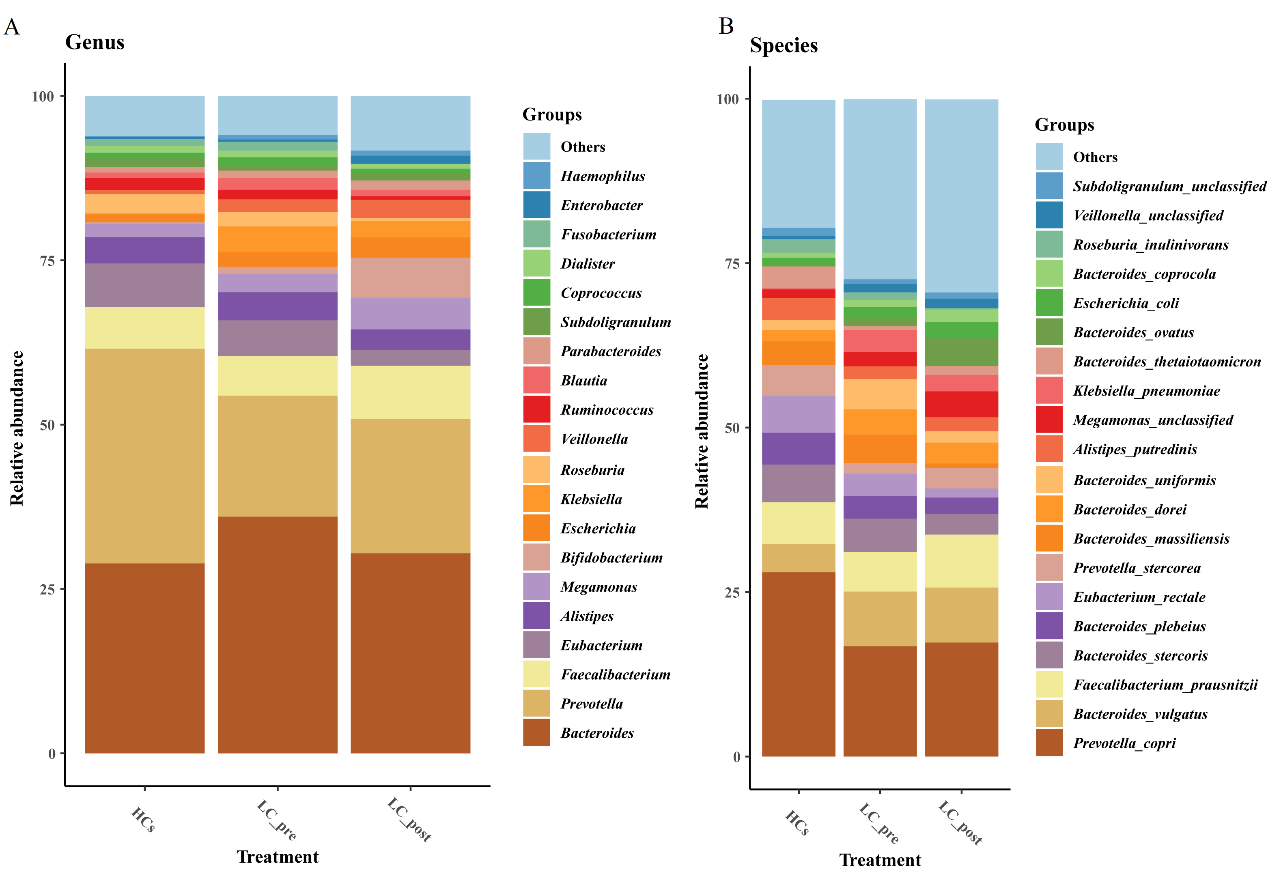


Supplementary Figure 3. Distribution and differences in abundances of Orthologous Groups (COG) categories in LC-pre (green), LC-post (blue), and HC groups (red) by the Wilcox test. Data were shown as box plots with the median and 25^th^ to 75^th^ percentiles. Benjamini- Hochberg correction was further applied to adjust derived p-values. Only pathways with p-values under a threshold of 0.05 were considered as significant.


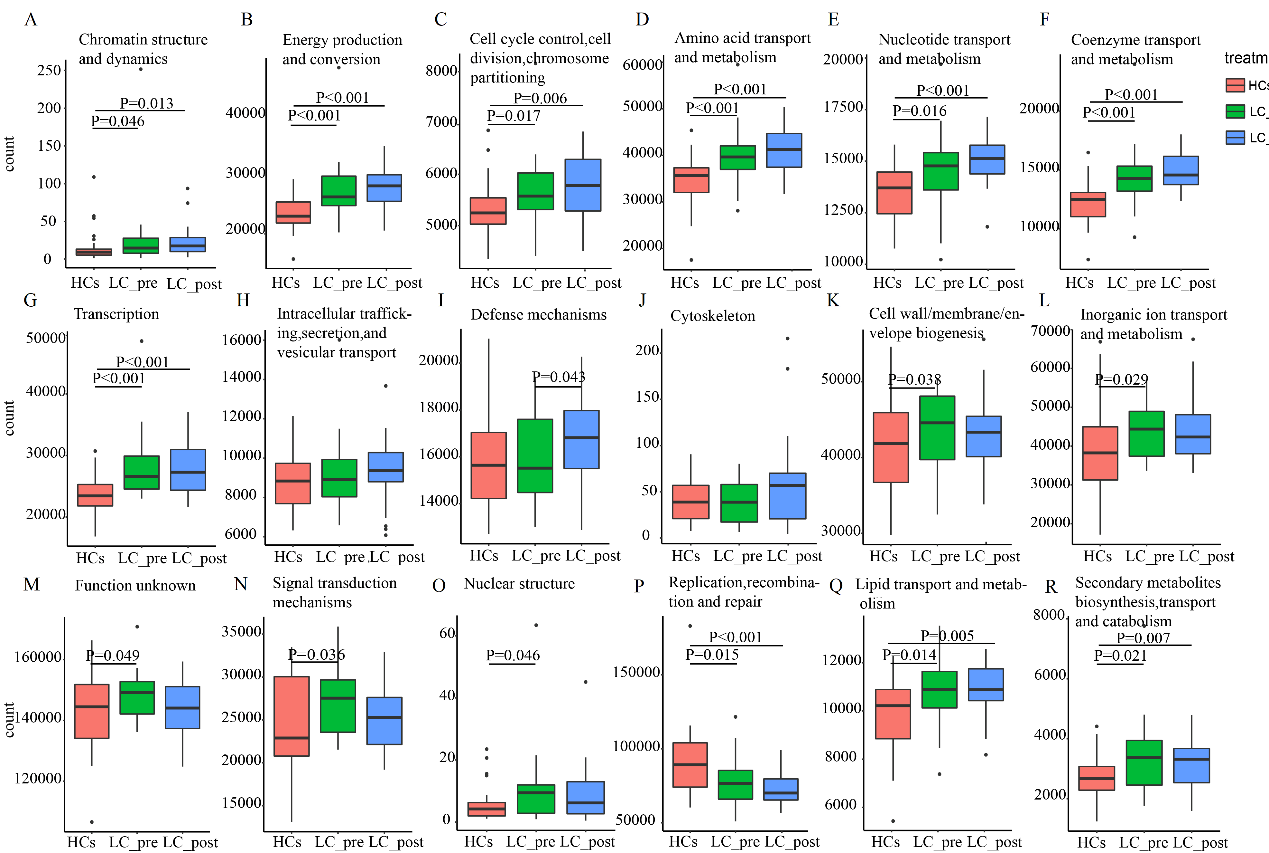


Supplementary Figure 4. Volcano plots comparing carbohydrate-active enzymes (CAZy) in fecal microbiome from subjects in group LC_pre versus HCs (A), LC_post versus HCs (B), and LC_pre versus LC_post (C). Genes were highlighted in red (upregulated) or blue (downregulated). GHs, glycoside hydrolases; GTs, glycosyl transferases; PLs, polysaccharide lyases; CEs, Carbohydrate esterases; and CBMs, Carbohydrate binding modules.


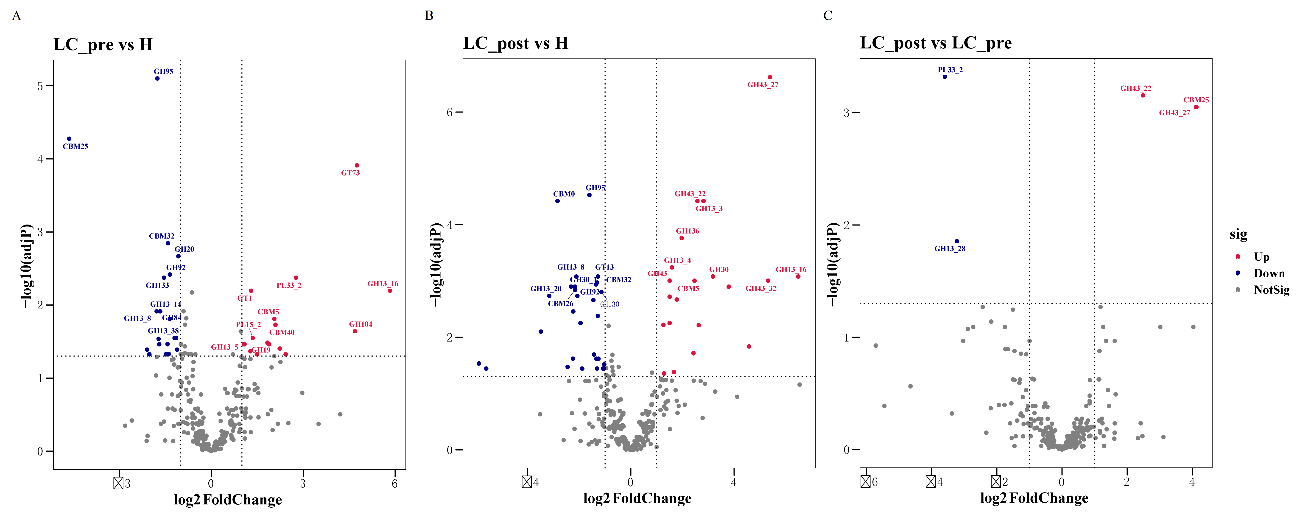


Supplementary Figure 5. Effect of lactitol on the β diversity of fecal BAs and SCFAs assessed by OPLS-DA.


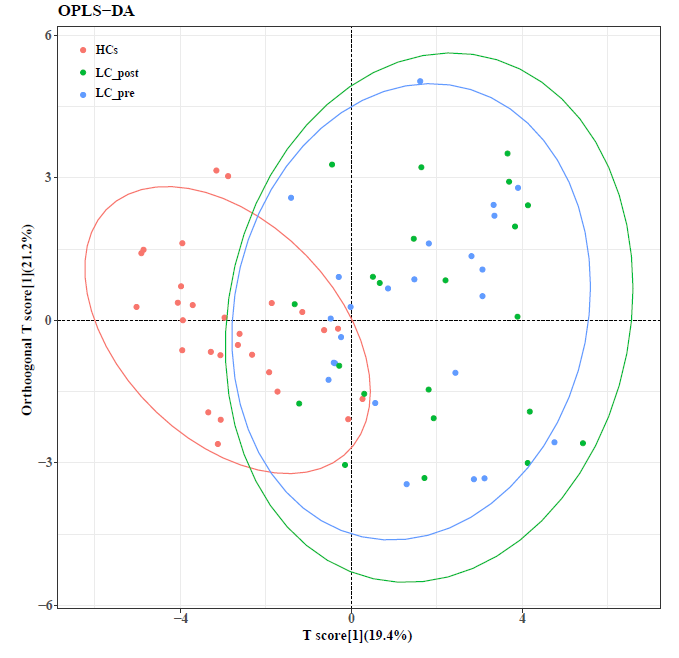

Supplement: Supplementary file 6 [file Data_Sheet_1.docx]
